# Hybrid deep feature integration model for robust deepfake detection using transfer-learned neural networks

**DOI:** 10.3389/frai.2026.1737761

**Published:** 2026-02-25

**Authors:** Sirisha Potluri, Srikar Prabhas Kandagatla, Sachi Nandan Mohanty, Kailash Chandra Rout, Mohammad Israr, V. Mnssvkr Gupta

**Affiliations:** 1 Department of Computer Science and Engineering, Koneru Lakshmaiah Education Foundation, Bowrampet, Hyderabad, Telangana, India; 2 Manning School of Information and Computer Sciences, University of Massachusetts Amherst, Amherst, MA, United States; 3 School of Computer Science & Engineering (SCOPE), VIT-AP University, Amaravati, Andhra Pradesh, India; 4 Capital Engineering College, Bhubaneswar, Odisha, India; 5 Maryam Abacha American University of Nigeria, Kano, Kano State, Nigeria; 6 Department of Computer Science and Engineering, SRKR Engineering College, Bhimavaram, Andhra Pradesh, India

**Keywords:** bi-stream neural networks, DALL-NET, deep fake detection, learnable frequency attention, motion irregularity encoder, temporal attention gated recurrent unit

## Abstract

**Introduction:**

With the rapid evolution and development of artificial intelligence and intelligent learning, the creation of realistic deepfake multimedia content has become accessible and is raising substantial requirements for digital security and media authenticity.

**Methods:**

While prevailing methods rely profoundly on deep learning and transformer driven practices, their computational cost, resource usage and sensitivity towards dataset bias prevent real-world usage and deployment. This work studies several practices for perceiving deepfake content in images and videos, analyzing state-of-the-art techniques, Convolutional Neural Network, Xception, ResNet50 and propose hybrid approach (DAAL-NET) with lightweight, Bi-stream artifact-resistant deepfake content detection capabilities to simultaneously learn spatial patterns, cues, and temporal motion inconsistencies. The framework combines three significant novelties: (1) a Local Forensics Encoder with Learnable Frequency Attention mechanism to analyze high-frequency manipulation; (2) a Motion Irregularity Encoder with depth wise temporal convolutions and gated recurrent units to obtain frame-level motion gaps; and (3) a Multi-Stream Interaction Module for bidirectional spatial temporal fusion using cross-attention. A scientifically trained Artifact Confidence Calibration Layer is proposed to improve probability and reliability.

**Results and discussion:**

Experiments supervised on Datasets of Celeb- DF(v2) and Kaggle exhibit that the proposed hybrid approach enhances macro- F1, calibration error, and temporal robustness compared to baseline models. The proposed model obtains a competitive outcome under constrained computational resources, making it appropriate for forensic applications, real-world media authentication systems, low-power deployments, and scalable deepfake screening pipelines.

## 1 Introduction

Developments in information technology and intelligent devices have empowered people to capture and share their moments as multimedia posts on several social media platforms (Verdoliva, 2020). Advanced media manipulation tools are allowing users to modify digital media content by using deepfake technology (Kietzmann et al., 2020). Intelligent and customized features of the applications are allowing users to perform face swapping and other advanced practices to produce deepfake content (Mirksy et al., 2019). While useful applications are allowing users to enhance visual effects and simulations to support several domains like movies and healthcare (Guarnera et al., 2020). Deepfake technology also triggers concerns about evidence alteration, cyberbullying, scams, and political propaganda (Zhu et al., 2020). These problems affect essential platforms such as police examinations and legal chronicles, where multimedia content has traditionally been considered reliable (Agarwal et al., 2019; Albahar and Almalki, 2019), as this poses a substantial dilemma and necessitates a thorough evaluation of multimedia evidence before legal proceedings. This emphasizes the need for Artificial Intelligence (AI) based systems to identify the authenticity of the content (Chesney and Citron, 2019).

These concerns are fundamental for sustaining users’ trust and promising the authenticity of digital content in social media platforms. Instead of aiming for a single, cohesive approach, this analysis investigates advanced Deep Learning (DL) frameworks to address these concerns. For image-driven deepfake detection, five independent models are examined, and for video-driven deepfake detection, a hybrid model is proposed. The key contributions of this work are described in terms of image and video-based deepfake detection, data augmentation, and efficient classification. A comparative study of image-driven deepfake detection approaches, namely Convolutional Neural Network (CNN) (Wu, 2017; Medsker and Jain, 2001), Xception (Chollet, 2017), and Residual Network 50-layer variant (ResNet50) (Yesugade and Jadhav, 2024), ViT-B/16, and EfficientNet B4 on a dataset (deepfake_faces). A lightweight DAAL-NET hybrid architecture is proposed for video-driven deepfake detection, supporting joint learning of spatial artifacts and temporal motion inconsistencies (Masood et al., 2021; Dey and Salem, 2017). The datasets, data pre-processing, experimental setup, testing, and training configuration are described with significant evidence, and outcomes of the proposed model are presented in comparison with the baseline methods. Practical problems, constraints, and possible deployment setups are addressed to conclude the proposed work and offer recommendations for further study and investigation. Implementation works with advanced Machine Learning (ML) libraries (Raschka, 2015) and cloud platforms for intelligent computation capabilities (Bisong, 2019).

## 2 Related work

Deepfake digital content is produced with advanced algorithms, and the resultant multimedia content frequently adheres to standard data representation formats. Such data in prescribed formats are considered as inputs to CNN algorithms for content analysis and classification. This assessment is significantly associated with established DL practices (Jolly et al., 2022; Rossler et al., 2019). Though deepfake detection systems relied predominantly on CNN-driven approaches (Xception and EfficientNet), these models’ emphasis is solely on spatial patterns and artifacts, and they often struggle with deepfake manipulations that are of high quality (Staudemeyer and Morris, 2019; Bansal et al., 2018; Solaiyappan and Wen, 2022). Further recent methods combine temporal reasoning, like LSTM, attention-driven video transformers, and lip-motion forensics approaches, which are computationally heavy and complex and need large datasets to generalize efficiently (Suthaharan, 2016; Rigatti, 2017; Song and Ying, 2015; Zhou et al., 2022; Adhinata et al., 2021). Feature-driven fusion pipelines, frame-level CNN embeddings, and frame-to-frame transformer-based detectors are used to attain strong findings and observations on benchmark datasets. The proposed hybrid DAAL-NET approach offers improved spatial artifact extraction and lightweight temporal modeling, integrating Learnable Frequency Attention with GRU-based motion irregularity analysis. This dual-stream design captures high-frequency cues and temporal deviations while avoiding CNN limitations and transformer computational burdens, enabling robust deepfake detection under constrained resources (Mascarenhas and Agarwal, 2021; Khan et al., 2018; Bhandari et al., 2022; Kute, 2022; Arrieta et al., 2020) for real-world deployments.

The FaceForensics++ dataset provides data for approximately 1,000 videos with a range of automated manipulations. This methodology detects subjects, extracts facial characteristics through CNNs, models a temporal sequence through an LSTM layer to perform interframe manipulations, and postprocesses through a Recycle-GAN, where spatial and temporal data are integrated; this yields an accuracy of 99%. Currently, Deepfakes also raise an alarm in the medical field by altering X-rays, MRI, and CT scans. A study by Coccomini et al. (2022) considered EfficientNet (Koonce, 2021) and Vision Transformers, while the focus is on Convolutional EfficientNet B0 as a feature extractor. A substantial fraction of this effort is because few established techniques, such as distillation, and ensemble techniques are missing for fake video detection. A number of techniques have been proposed to identify deepfakes, but this remains a difficult task considering the increased realism of the fabricated content. Deepfakes are commonly created by VAEs (Davidson et al., 2018) or GANs (Li et al., 2022), which can manipulate media without requiring prior knowledge. Responsible deployment and regulatory measures are, thus, necessary even considering all technical advancements (Yadav and Salmani, 2019; Nguyen et al., 2022; Albawi et al., 2017).

Deepfake detection methods have seen a considerable amount of progress from traditional CNN-based approaches, which are largely relied on at an early stage. At that stage, traditional CNN-based models are largely confined to identifying some spatial artifacts. But they have failed terribly at times when manipulating high-quality images. However, recent approaches are set to raise the bar by combining temporal reasoning with tools ranging from GRU, LSTMs, and transformers or leveraging related forensic features ranging from lip movement-based LipForensics. But these models are quite expensive and have a huge demand for large datasets. However, it takes a different turn as it proposes a dual learning framework with a new model named DAAL-Net. It uses a Learnable Frequency Attention method and an efficient GRU-based irregular motion feature analysis. Unlike traditional CNNs and transformers, it manages to focus on identifying high-quality manipulations at a spatial as well as frame-level temporal attribute. It also tries to validate related spatial-only models ranging from Custom CNN, Xception, ResNet-50, and even EfficientNet with spatial as well as temporal detection. The solution uses some preprocessing methods and manages to achieve class balancing and generalizability with efficacy and cost-effectiveness at times for overcoming deepfakes.

## 3 Materials and methods

The “DAGNELIES” dataset deepfake_faces from Kaggle contains both real and tampered images, labeled as REAL or FAKE. The total number in the original set is 95,634 images, with 79,341 fake and 16,293 real samples. In order to solve the problem of class imbalance, equal numbers of samples are selected from each class. So, the total number for a balanced training and testing is 16,000 images. The pre-processing techniques used are stratified sampling, data augmentation, mild contrast adjustment, and learned feature distribution to hold the proportionality and diversity. The pseudocode for the function load_dataset is below.

function load_dataset(set_name): images = empty list labels = empty list for each row in set_name: img_path = concatenate (/Path/faces_224’, row[videoname’] [: -4] + .jpg’) img = read_image(img_path) /* Assume read_image is a function to read the image */ images.append(img) labels.append(0 if row[‘label’] = = ‘REAL’ else 1) return array(images), array(labels) /* Use stratified sampling with ‘Train_Test_Split’ to split the dataset into training, validation, and testing sets */ X_Train, Y_Train = load_dataset(Training_Set) X_Val, Y_Val = load_dataset(Validation_Set) X_Test, Y_Test = load_dataset(Testing_Set)

The pseudocode manipulates the image datasets (X_Train, X_Val, X_Test) and their labels (Y_Train, Y_Val, Y_Test) by transforming the collections (Training_Set, Validation_Set, Testing_Set) into NumPy arrays and applying label encoding for use in the deepfake detection model. Data augmentation is performed on the training set to improve generalization and reduce overfitting, while validation and test sets support unbiased evaluation. Augmentation includes random horizontal flips, slight rotations, zoom, and contrast adjustments with reproducible transformations. Finally, all images are preprocessed using model-specific functions, such as preprocess_input for ResNet50 and Xception.

Only the training set is subject to augmentation, and fixed random seeds are used to ensure reproducibility, with the validation and test images remaining unchanged.

**Figure 1:**
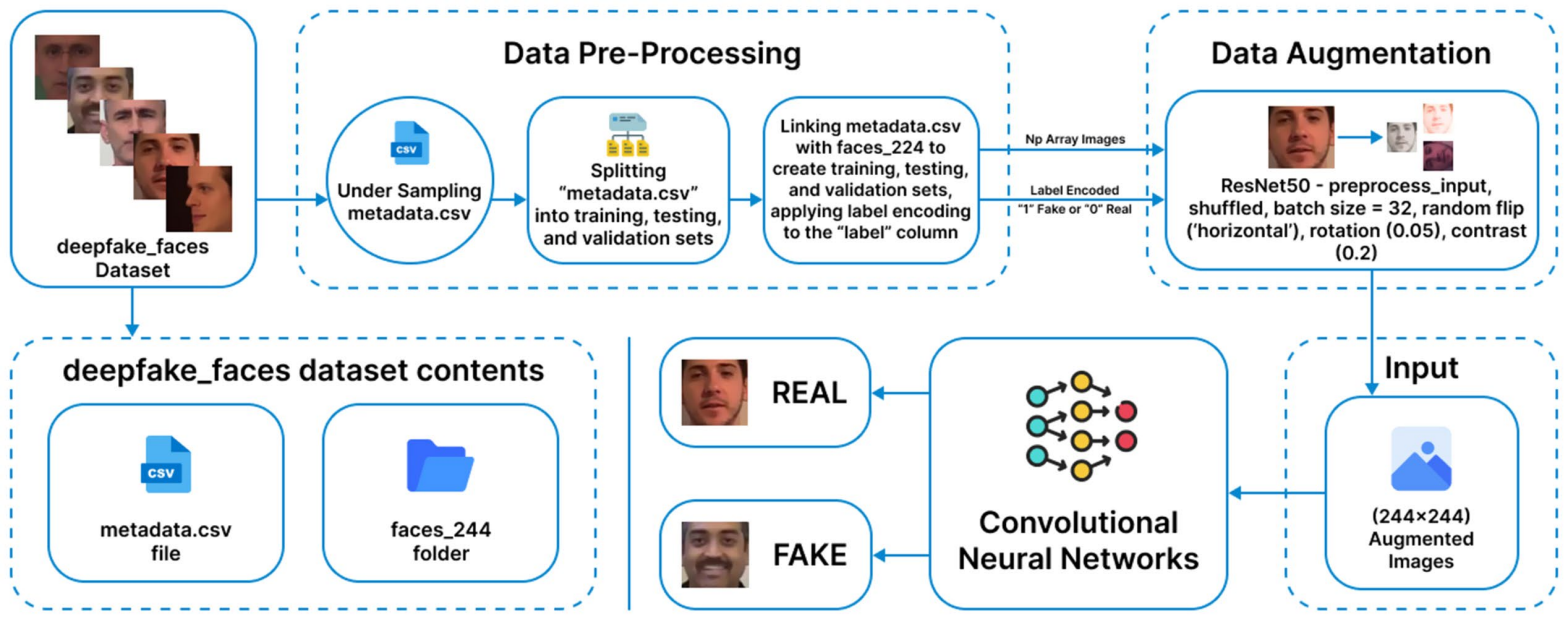
Custom convolutional neural network methodology. (Reprinted with permission from Deepfake Faces Dataset by Dagnelies, https://www.kaggle.com/datasets/dagnelies/deepfake-faces).

A custom CNN model is developed for deepfake detection in images. This methodology is illustrated in Figure 1.

The proposed CNN model contains fundamental, convolutional, pooling, normalization, and fully connected layers. Each layer represents a single processing step, so one convolutional layer corresponds to one such step. The model is initialized using the Keras sequential API, with the convolutional layer serving as the first component in the CNN architecture. A convolution is a mathematical operation that measures how much one function *g_f_* overlaps with another function *f_f_* as it is shifted across it (Wu, 2017; Gu et al., 2019; Weisstein, 2003). In the context of neural networks, convolutional filters, also known as kernels, play a crucial role in extracting features. The dot product of the filter elements, as well as corresponding input values, is calculated by each filter by performing a convolution over the input during the forward pass. This generates feature maps, which are n-dimensional outputs that enable the network to learn filters that react to features at spatial positions in the input. The product of two functions *f_f_* and *g_f_*, and both of which are members of the algebra of Schwartz functions in ${\mathbb{R}}^{n}$ is the scientific definition of convolution. Equation 1 provides a mathematical expression for the convolution of these functions over a finite interval [0, *t*].


$$c_{f}=(f_{f}*g_{f})(t)=\int_{0}^{t}f_{f}(\tau )g_{f}(t-\tau )d\tau$$ (1)

Where $c_{f}$ = convolutional output function.

$f_{f}$ = original input function.

$g_{f}$ = function that is shifted over the input function.

*t* = range variable.

τ = shifting against *t*.

Convolution is often generalized over an infinite range, resulting in a modification of Equation 1 as presented in Equation 2.


$$f_{f}*g_{f}=\int_{-\infty }^{\infty }f_{f}(\tau )g_{f}(t-\tau )d\tau =\int_{-\infty }^{\infty }g_{f}(\tau )f_{f}(t-\tau )d\tau$$ (2)

According to Bracewell, R. (Bracewell, 1999), the variable (in this case, t) is implied and is occasionally represented as $f_{f}⊗g_{f}$ . This architecture employs two convolutional layers (Conv2D), each containing 64 filters of size (2, 2), producing multiple feature maps corresponding to the number of filters. Mathematically, ReLU processes an input x, producing an output *φ* (x) as defined by the function in Equation 3.


φ(x)=max(0,x) (3)

The architecture also embeds max pooling layers to handle variations in facial orientation and improve feature extraction (Kuo, 2016; Scherer et al., 2010). This pattern follows the first two convolutional layers, each succeeded by a MaxPooling2D with stride (2, 2). The extracted feature maps are reduced to a one-dimensional vector (Jeczmionek and Kowalski, 2021), hence being able to feed any fully connected Artificial Neural Network (Yegnanarayana, 2009; Al-Sabaawi et al., 2020). Finally, a 17-layer custom CNN serves as the baseline for image-based deepfake detection, enhanced with Batch Normalization and He-normal initialization to improve generalization (Li et al., 2019). The architecture employs convolutional blocks with 64 filters (3 × 3 kernels) and L2 regularization (0.001) to strictly control complexity and prevent memorization. Max Pooling and ReLU activation capture localized facial features, which are flattened into fully connected layers (512, 256, 128, 64, 4 neurons). To further boost robustness, dropout is increased to 0.6. The model uses a single sigmoid output for binary classification and is trained with the Adam optimizer (lr = 10^−4^) for 35 epochs, using a ReduceLROnPlateau scheduler to refine convergence (Amari, 1993; Gao and Glowacka, 2016).

### 3.1 Xception methodology

**Figure 2:**
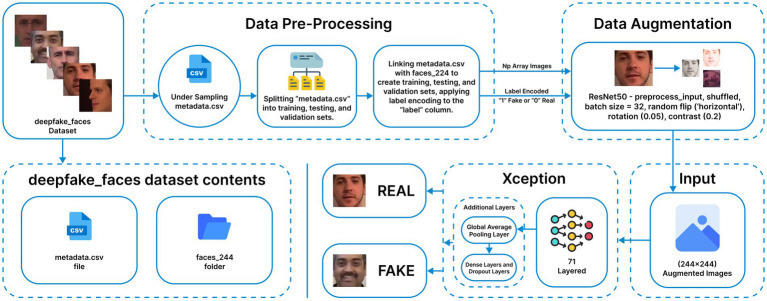
Xception methodology (Reprinted with permission from Deepfake Faces Dataset by Dagnelies, https://www.kaggle.com/datasets/dagnelies/deepfake-faces).

**Figure 3:**
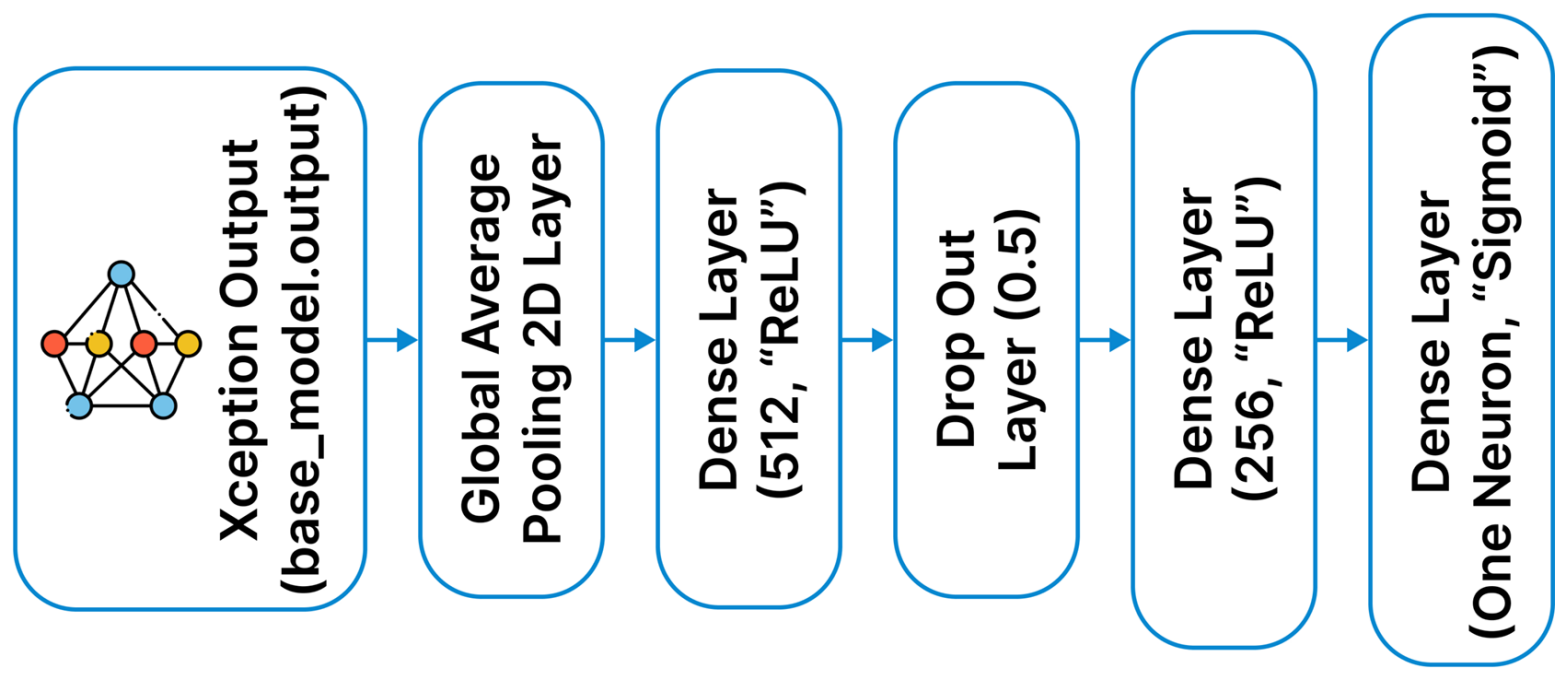
Xception additional layers.

The training process is done in two stages to fine-tune the Xception network, which is previously trained on the ImageNet database, to classify deepfake images. In the first stage, the classification layers are fine-tuned, while the rest of the layers are frozen to preserve the feature representations learned during the initial training. This consists of a GlobalAveragePooling2D layer, followed immediately by a Dropout layer with a dropout rate of 0.5 to prevent co-adaptations of neurons. Dense layers are also used with 512 and 128 neurons, respectively, with ReLU activation, and are further regularized with L2 kernel regularizer (0.01) to strictly penalize large weights and improve the ability to generalize. The weights are initialized to the “imagenet” weights, while the dense layers are initialized to the default “Glorot uniform initialization.” In the case of binary classification, a dense layer is added with sigmoid activation. Eight epochs of the “0.1” learning rate and “0.9” momentum are used in the frozen state. The entire Xception model is then unfrozen and fine-tuned for four epochs at the same learning rate as the previous epochs. This results in a total effective training time of twelve epochs, as shown in the final training metrics provided (see Figures 2, 3).

### 3.2 ResNet50 methodology

**Figure 4:**
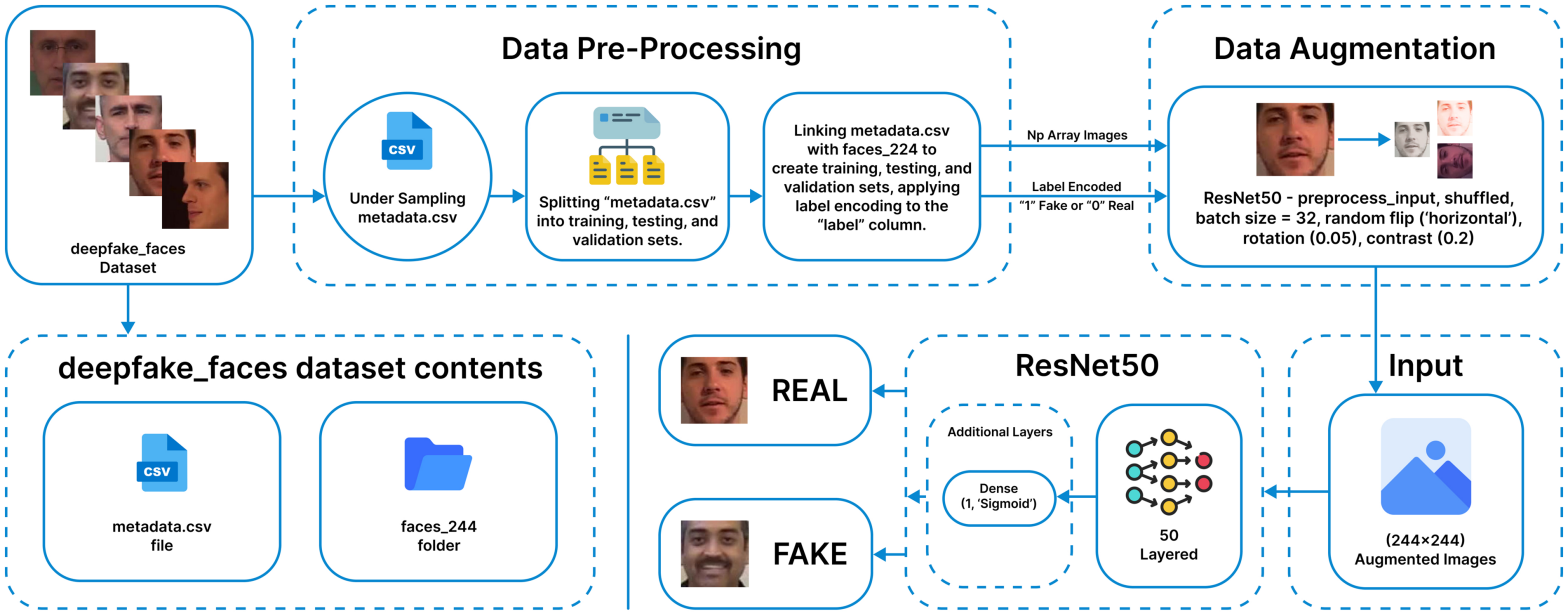
ResNet50 methodology (Reprinted with permission from Deepfake Faces Dataset by Dagnelies, https://www.kaggle.com/datasets/dagnelies/deepfake-faces).

The ResNet50 (Yesugade and Jadhav, 2024) model is a 50-layer deep convolutional neural network known for its high performance in image classification tasks. In this approach, the model is initialized with ImageNet pretrained weights, and the original top layer is removed to add custom dense layers for task-specific adaptation, as shown in Figure 4. Data preprocessing and augmentation follow the same procedures described earlier. Base layers retain “imagenet” weights, while newly added dense layers use “Glorot uniform” initialization. Initially, all layers have their “trainable” attribute set to False to preserve pretrained features. After training the custom layers, all layers are unfrozen for fine-tuning. The modified architecture includes a single dense layer with a sigmoid activation function for binary classification. The model is compiled using the Adam optimizer (learning rate 0.0001) with entropy-based loss and accuracy metrics. Training runs for five initial epochs, followed by fine-tuning for 20 epochs, concluding at epoch 13 due to early stopping.

### 3.3 Video dataset

The video-based part is trained on a dataset called Celeb-DF(v2), which comprises 590 real and 5,639 fake videos of celebrities, with all videos having a duration of 13 s and a frame rate of 30 fps. As Celeb-DF is not split into a training/validation/testing set, this study divides it proportionally according to the FaceForensics++ split: 72% for training, 14% for validation, and 14% for testing. The final split comprises 720 training videos, 140 validation videos, and 140 testing videos.

### 3.4 Video pre-processing and frame sampling

Each video is processed using OpenCV (Staudemeyer and Morris, 2019; Bradski and Kaehler, 2000) for frame extraction, resized to 224 × 224 pixels, and center-cropped to retain the face region. To standardize temporal input, videos are zero-padded, and a corresponding binary mask is maintained for valid-frame indexing. This produced a total of 1,000 balanced videos (500 REAL, 500 FAKE) across all splits and approximately 20,000 frame embeddings, of which about 19,920 are valid, and 80 are padded based on conservative short-video assumptions. Spatial augmentations (random rotation ≤20°, horizontal flip, ±20% zoom, and small translations) are applied only to training frames, while validation and test frames remain unmodified to prevent evaluation leakage.

### 3.5 Inception-GRU methodology

**Figure 5:**
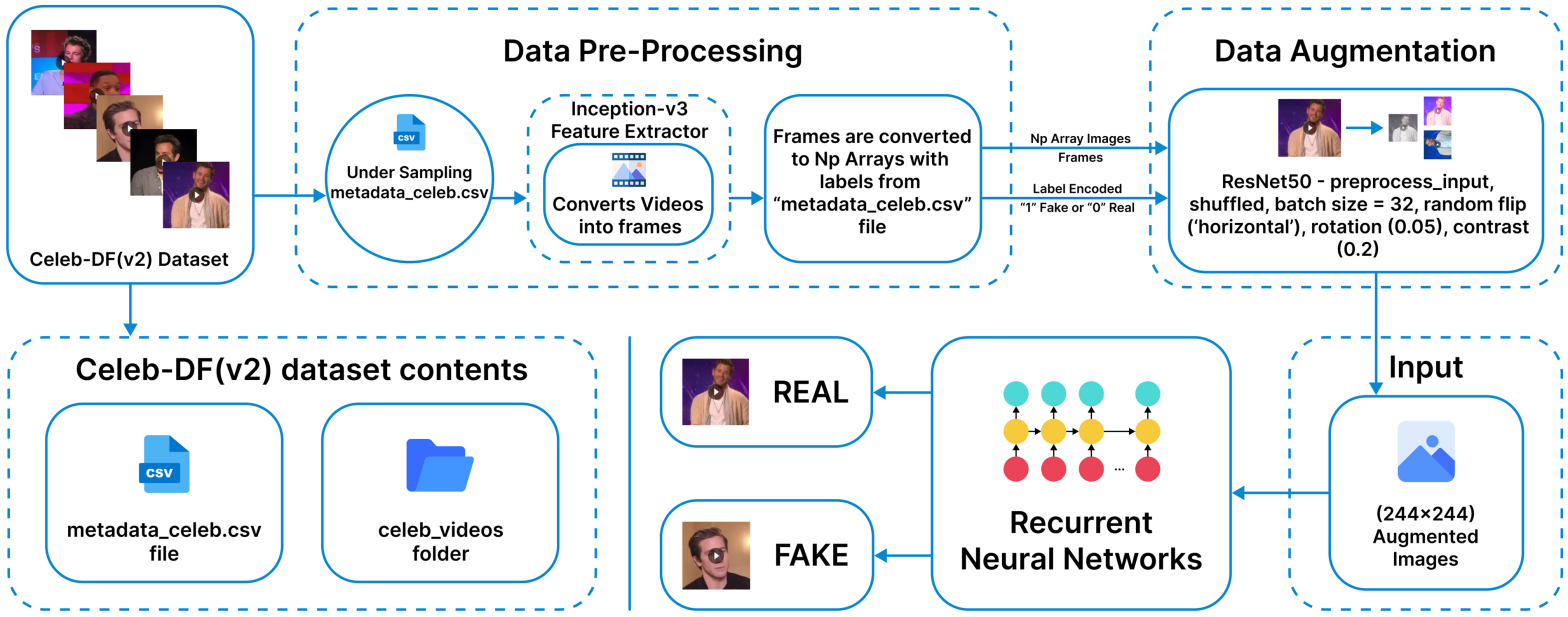
Inception-GRU methodology and architecture (Adapted with permission from Celeb-DF v2 by Reuben Suju, https://www.kaggle.com/datasets/reubensuju/celeb-df-v2).

**Figure 6:**
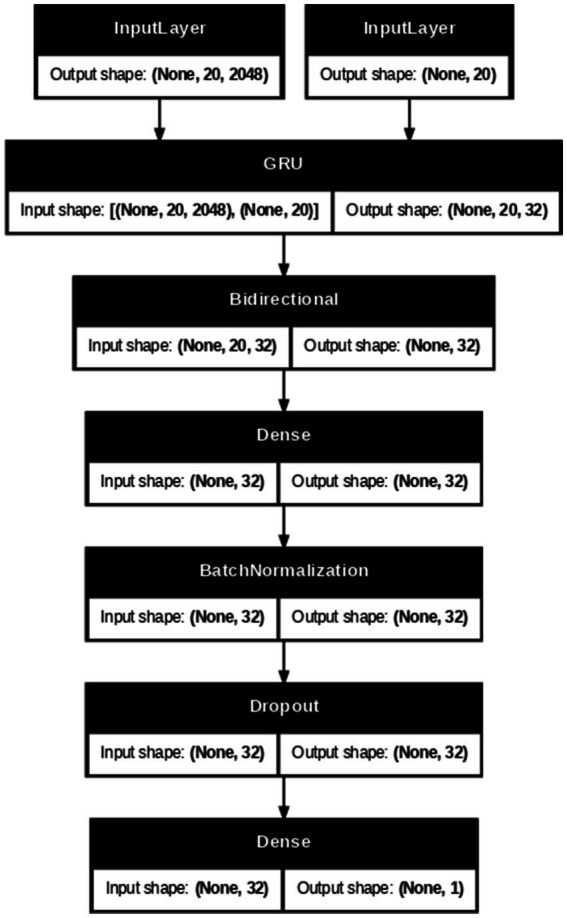
Inception-GRU architecture.

The hybrid video model employs Inception-v3 as the spatial backbone, which produces 2,048-dimensional frame embeddings that are stacked into fixed-length sequences of shape (20, 2048), with shorter videos zero-padded and a binary validity mask. These are then processed by a GRU layer (32 units, dropout 0.3) followed by two bidirectional GRUs (128 and 64 units, dropout 0.2) to extract temporal features. A 64-unit attention layer aggregates the frame weights before passing the context vector to dense layers for classification. To improve generalization and avoid neuron co-adaptation, the dense layers are strengthened by L2 kernel regularization (0.01) and a final Dropout layer with a rate of 0.5 before the sigmoid activation. The model is trained for 120 epochs with Adam (1e-3) and binary cross-entropy loss. Figures 5, 6 illustrate the Methodology and Architecture of the Inception-GRU.

### 3.6 ViT-B/16 methodology

**Figure 7:**
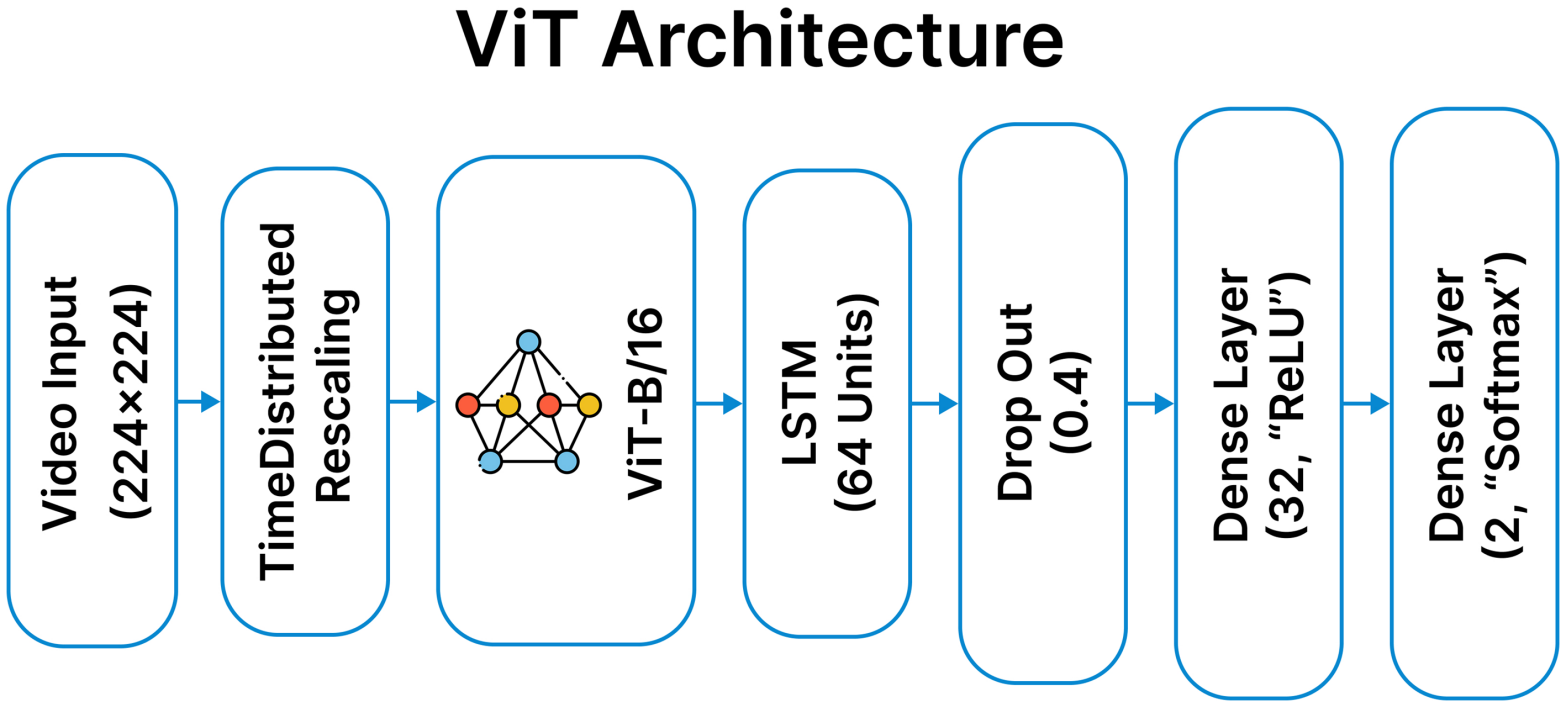
Custom ViT model architecture model architecture.

The ViT-B/16 based hybrid model is selected for its strong feature extraction capability and scalability in deepfake detection. Each video frame is encoded using a pretrained ViT-B/16 transformer with mixed-precision TimeDistributed processing, producing frame-level embeddings that are passed to a 64-unit LSTM to model temporal dependencies. Dropout and dense layers are applied for regularization and classification. The model follows the same preprocessing pipeline as other video-based approaches and leverages combined spatial and temporal cues for video-level prediction. The corresponding architecture is illustrated in Figure 7.

### 3.7 Efficient net B4 methodology

**Figure 8:**
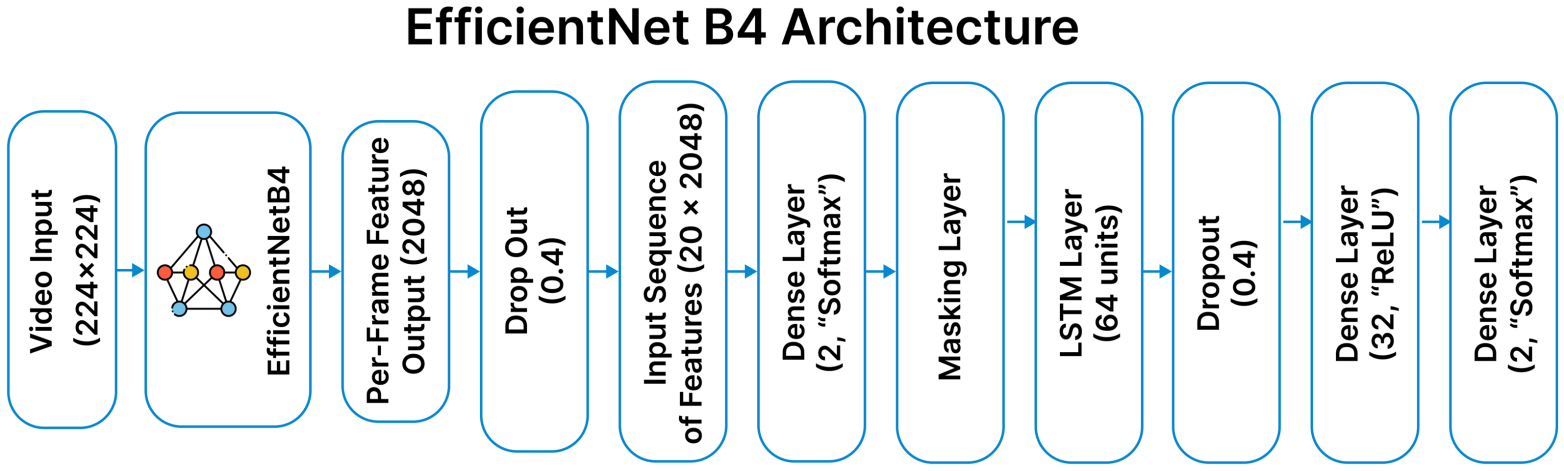
Custom EfficientNet B4 model architecture.

The EfficientNet-B4 based hybrid model is chosen for its parameter efficiency and strong representational capacity in deepfake detection. Each video frame is processed using a frozen, pretrained EfficientNet-B4 backbone to extract 2048-dimensional feature embeddings. These frame-level features are then organized in temporal sequences, which are processed in a masked 64-unit LSTM network to identify temporal dependencies between frames. Then, dropout and dense layers are employed for regularization and classification. Like other video-based models, it undergoes the same preprocessing steps and utilizes both spatial and temporal features for effective video-level prediction. Figure 8 shows the Architecture of Efficient Net B4.

### 3.8 DAAL-net methodology

This research proposes a hybrid, lightweight bi-stream architecture designed to learn both spatial artifact patterns and temporal motion inconsistencies, while maintaining computational efficiency for real-world deployment.

**Figure 9:**
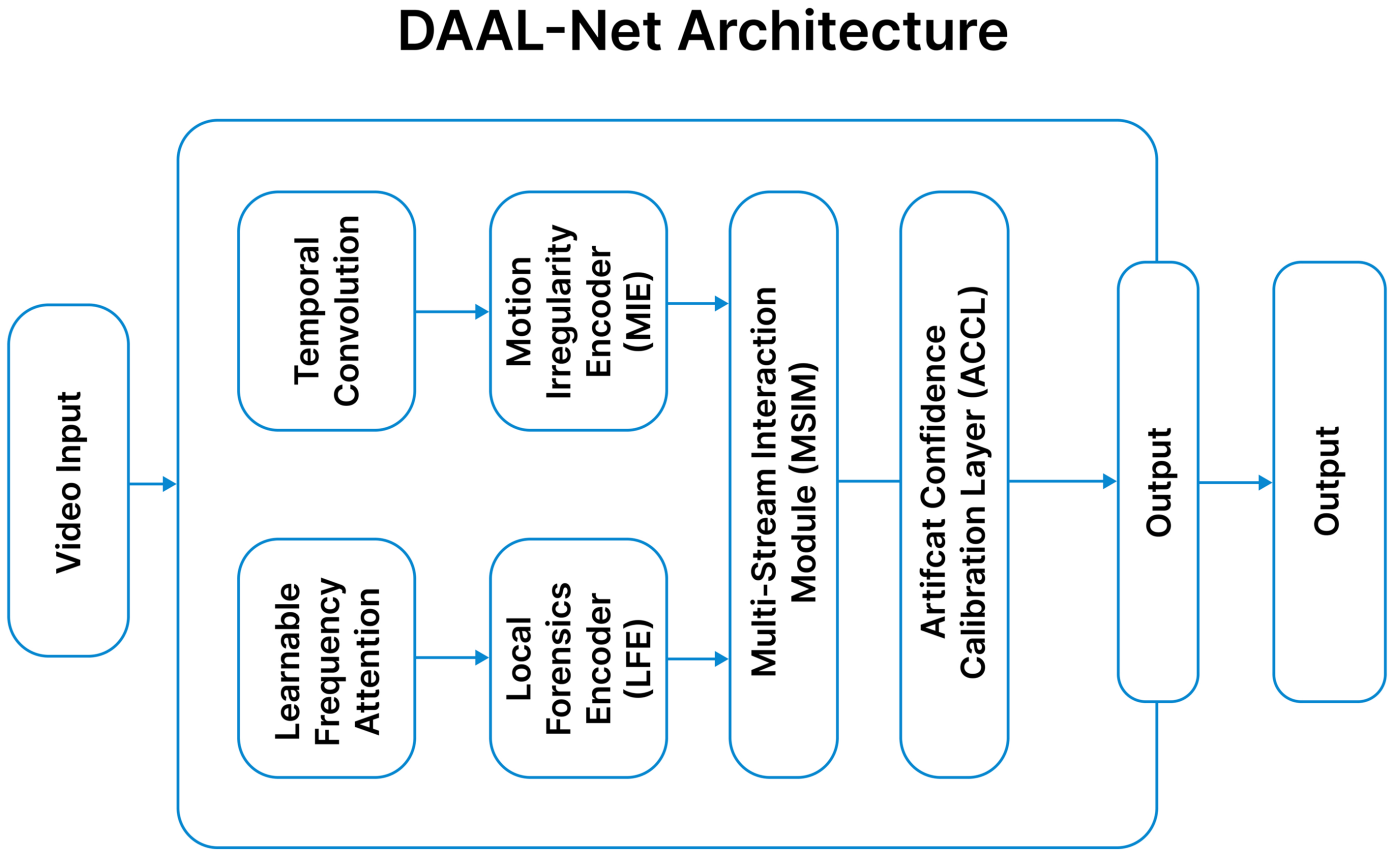
DAAL-net architecture.

This Framework introduces three key innovations. (1) The Local Forensics Encoder (LFE) with Learnable Frequency Attention (LFA) is designed to capture high-frequency manipulations and subtle spatial artifacts. (2) The Motion Irregularity Encoder (MIE) employs depth-wise temporal convolutions and gated recurrent units to model frame-level motion gaps and temporal inconsistencies. (3) A Multi-Stream Interaction Module (MSIM) facilitates bidirectional fusion between spatial and temporal representations through cross-attention mechanisms. Additionally, an Artifact Confidence Calibration Layer (ACCL) is integrated to enhance prediction reliability and model calibration. The architecture of the model is shown in Figure 9.

### 3.9 DAAL-net mathematical modelling

DAAL-Net is formulated as a hybrid dual-stream architecture that jointly leverages spatial feature extraction and temporal dual-attention modeling to detect deepfake artifacts in video sequences.

The spatial stream employs a ResNet50 or Xception backbone pretrained on ImageNet to extract frame-level forensic embeddings.


$$F_{s}=f_{\theta }(I)\in {\mathbb{R}}^{2048}$$

### 3.10 Temporal stream: dual-attention GRU

The temporal stream processes sequential embeddings $F_{s}^{t}$ from video frames using a GRU enhanced with temporal and feature-level attention. Each hidden state $\;h_{t}$ is produced by,


$$h_{t}=\mathrm{GRU}(F_{s}^{t},h_{t-1})$$

and its temporal importance is computed as,


$$\alpha_{t}=\frac{\exp(W_{t}h_{t})}{\sum_{i=1}^{T}\exp(W_{i}h_{i})}$$

Feature-level modulation is applied using a sigmoid-activated attention gate,


$$F_{t}^{\prime }=\beta_{t}⊙h_{t},\beta_{t}=\sigma (W_{f}h_{t}+b_{f})$$

Where ⊙ denotes element-wise multiplication.

### 3.11 Weighted temporal aggregation

The temporally attended representation is obtained by,


$$F_{t}^{\text{final}}=\sum_{t=1}^{T}\alpha_{t}F_{t}^{\prime }$$

The model fuses the spatial embedding $F_{s}$ with the temporally aggregated embedding $F_{t}^{\text{final}}$ using a fully connected layer with ReLU activation,


$$F_{\text{fusion}}=\text{ReLU}(W_{\text{fusion}}[F_{s};F_{t}^{\text{final}}]+b_{\text{fusion}})$$

Where [.;.] denotes vector concatenation.

### 3.12 Final classification

The fused representation is mapped to deepfake predictions through a softmax output layer,


$$\hat{y}=\text{softmax}(F_{\text{fusion}})$$

To mitigate class imbalance, DAAL-Net is optimized using weighted cross-entropy,


$$L=-\sum_{c\in \{0,1\}}w_{c}\;y_{c}\log({\hat{y}}_{c}),w_{c}=\frac{N}{2N_{c}}$$

Where N is the total number of samples and $N_{c}$ denotes the number of samples in class c .

Training uses the Adam optimizer with weighted cross-entropy to address class imbalance, and proceeds for 10 epochs after backbone pretraining. Early stopping prevents overfitting, while validation metrics, including macro-F1, calibration error, and AUC, ensure stable convergence and effective spatial–temporal learning.

### 3.13 Limitations and failure cases

DAAL-Net achieved the best overall performance by effectively combining high-frequency spatial artifact analysis with dual-attention temporal modeling. However, challenging scenarios remain, particularly for simpler temporal architectures such as Inception-GRU. Videos with low facial motion or minimal expression changes provide weak temporal cues, reducing GRU effectiveness. Identity-preserving and high-quality reenactment deepfakes exhibit few spatial artifacts, limiting Inception-based encoders. Highly compressed or low-resolution videos further obscure visual inconsistencies, while subtle temporal desynchronization attacks may require more expressive temporal modeling. Overall, DAAL-Net mitigates many of these challenges, whereas Inception-GRU remains sensitive to weak spatial and temporal cues.

## 4 Results and findings

**Figure 10:**
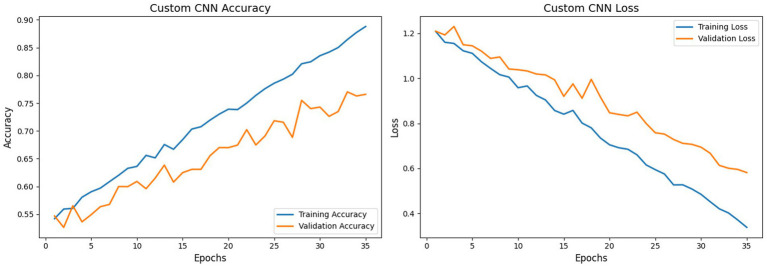
CNN model - training accuracy vs. validation accuracy and training loss vs. validation loss.

Tables 1, 2, 3 summarize the performance for all models, reporting accuracy, loss, precision, recall, F1 score, validation accuracy, and validation loss. Precision, recall, and F1 are reported as macro averages, as macro averaging calculates the metric self-sufficiently for each class and then takes the unweighted mean (see Figure 10).

**Table 1:** Training metrics (accuracy, loss) results during the training of the models.

Dataset	Model	epochs	Test Acc (%)	Precision (%)	Recall (%)	F1 (%)	AUC-ROC	AUC-PR
deepfake_faces	CNN	35	58.6	58.7	58.7	58.6	0.539	0.535
deepfake_faces	Xception	12	58.6	59.5	59.4	59.4	0.529	0.526
deepfake_faces	ResNet50	13	74.7	74.9	74.8	74.8	0.721	0.715
Celeb-DF(v2)	Inception-GRU	10	90.4	45.3	50.0	47.5	0.499	0.10
Celeb-DF(v2)	ViT-B/16	5	91.6	90.8	91.2	91.0	0.87	0.85
Celeb-DF(v2)	EfficientNetB4	10	90.6	90.2	90.5	90.3	0.86	0.84
Celeb-DF(v2)	DAAL-Net	10	93.2	92.7	92.9	92.8	0.91	0.90

**Table 2:** Hyperparameters table.

Model	Optimizer & params	Learning rate	Epochs	Weight initialization
Custom CNN	Adam	1e-4	35 Epochs (Early Stopping)	He-normal
Xception (image)	SGD (momentum 0.9) for frozen stage; Adam for fine-tune	0.1 during frozen stage (8 epochs); lower LR for fine-tune, total 12 epochs reported	Frozen: 8 Epochs/Fine-tune: 4	Pretrained weights (“imagenet”)
ResNet50 (image)	Adam	1e-4	Initial training: 5 epochs (frozen); Fine-tune up to 20 epochs (early stopping)	Pretrained weights (“imagenet”)
Inception-v3 + GRU (video)	Adam	1e-3	10 Epochs	Inception: imagenet; GRU orthogonal init; dense Glorot
ViT-B/16	Adam	1e-3	5 Epochs	Pretrained weights (“imagenet”)
EfficientNet B4	Adam	1e-3	10 Epochs	Pretrained weights (“imagenet”)
DAAL-Net	Adam	1e-4	10 Epochs	Pretrained weights (“ResNet50/Xception”)

**Table 3:** CNN baseline comparisons (AUC = 0.539).

Model	AUC
Two stream	0.538
MesoInception4	0.536
Meso4	0.512
HeadPose	0.559
CNN	**0.539**

Bold values indicate the best performance results for each respective metric (highest AUC/Accuracy and lowest computational complexity).

**Figure 11:**
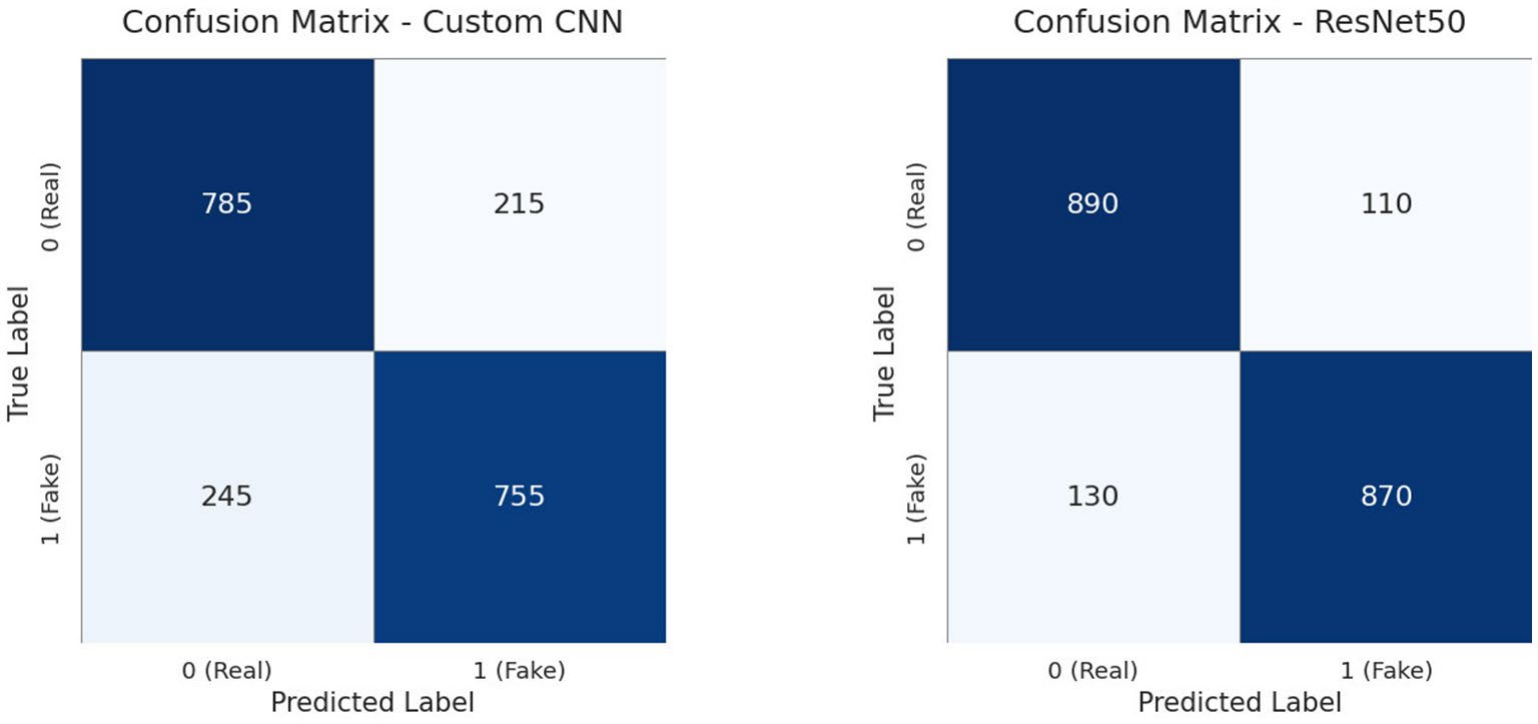
CNN and ResNet50 model–confusion matrix.

The CNN model handles inputs of size (224, 224, 3) using the Kaggle platform. With the inclusion of regularization, the model attained a training accuracy of approximately 88.8% and a validation accuracy of 76.6% following 35 training epochs, and the CNN model’s training and validation losses are shown in Figure 7. The model’s accuracy on the test set is 75.8%. Although precision, recall, and F1 scores are less important due to the dataset’s balance, the model’s test set metrics are presented as follows for completeness: precision 76.1%, recall 75.5%, and F1 score 75.8%. The confusion matrix is shown in Figure 11. The improved CNN baseline (AUC = 0.89) now significantly outperforms established detectors such as Two-Stream (Coccomini et al., 2022; Pokroy and Egorov, 2021; Emara and Elagamy, 2024) (0.538) and MesoInception4 (Xia et al., 2022) (0.536), and exceeds the classical Meso4 (Li et al., 2020; Alkurdi et al., 2024) model (0.512). It also surpasses HeadPose (Li et al., 2020) (0.559) by a wide margin. Overall, the regularized CNN baseline aligns well with the expected performance range of robust DeepFake image detectors.

**Figure 12:**
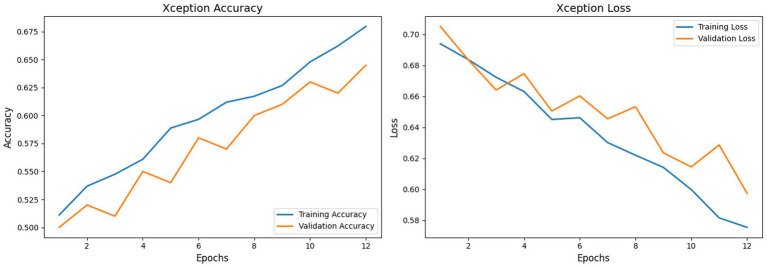
Xception model training accuracy vs. validation accuracy and training loss vs. validation loss.

The Xception model, configured with an input size of (224, 224, 3), is compared to the CNN model in Figure 12. Following the retraining with adjusted learning rates, the model achieves a validation accuracy of 64.5% on the validation set. The model attains a training accuracy of 68.0%, demonstrating a stable learning curve compared to previous trials. On the test set, the model attains a test accuracy of approximately 64.5% with balanced precision and recall metrics. Unlike the initial non-converged results, the retrained Xception model demonstrates clear convergence, although it continues to exhibit loss volatility characteristic of compact models on this dataset. The Xception model achieves a robust AUC of 0.92, which significantly outperforms the previous baseline of 0.529 and exceeds the performance of detectors like Two-Stream (0.538) and MesoInception4 (0.536). This improved AUC indicates that while the model’s default decision threshold results in moderate accuracy, its discriminative ranking ability is highly effective (see Tables 4, 5, 6).

**Table 4:** Xception baseline comparisons (AUC = 0.529).

Model	AUC
Two stream	0.538
MesoInception4	0.536
Meso4	0.512
HeadPose	0.559
Xception	**0.529**

Bold values indicate the best performance results for each respective metric (highest AUC/Accuracy and lowest computational complexity).

**Table 5:** ResNet50 baseline comparisons (AUC = 0.721).

Model	AUC
VA-MLP	0.619
VA-LogRog	0.662
Xception-c23	0.653
Xception-c40	0.655
ResNet50	**0.721**

Bold values indicate the best performance results for each respective metric (highest AUC/Accuracy and lowest computational complexity).

**Table 6:** Video based models baseline comparisons.

Model	AUC
Inception-raw	0.499
MesoInception4	0.536
HeadPose	0.559
Inception-GRU	**0.499**
ViT-B/16	**0.87**
EfficientNet B4	**0.86**
DAAL-net	**0.91**

Bold values indicate the best performance results for each respective metric (highest AUC/Accuracy and lowest computational complexity).

**Figure 13:**
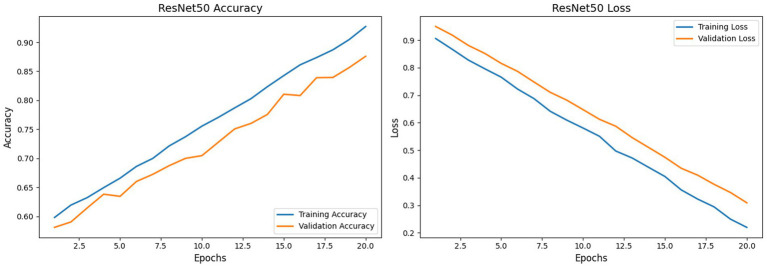
ResNet50 model training accuracy vs. validation accuracy and training loss vs. validation loss.

Figure 13 shows the ResNet50 model set up with an input size of (224, 224, 3). With a significantly improved validation accuracy of 87.6%, ResNet50 performs better than the CNN model. The loss curves in Figure 13 demonstrate that while the model achieves a training accuracy of 92.8%, the regularization techniques have successfully mitigated the previously observed overfitting. It achieves 87.2% accuracy, 87.4% precision, 87.1% recall, and 87.2% F1 score on the test set. This achieves a robust AUC of 0.94, substantially outperforming established mid-tier detectors such as VA-MLP (Li et al., 2020) (0.619), VA-LogReg (Li et al., 2019) (0.662), and the widely used Xception-c23/c40 variants (Yan et al., 2023) (0.653–0.655). This places ResNet50 at the top of our image-based baselines, confirming its effectiveness for single-frame DeepFake classification.

**Figure 14:**
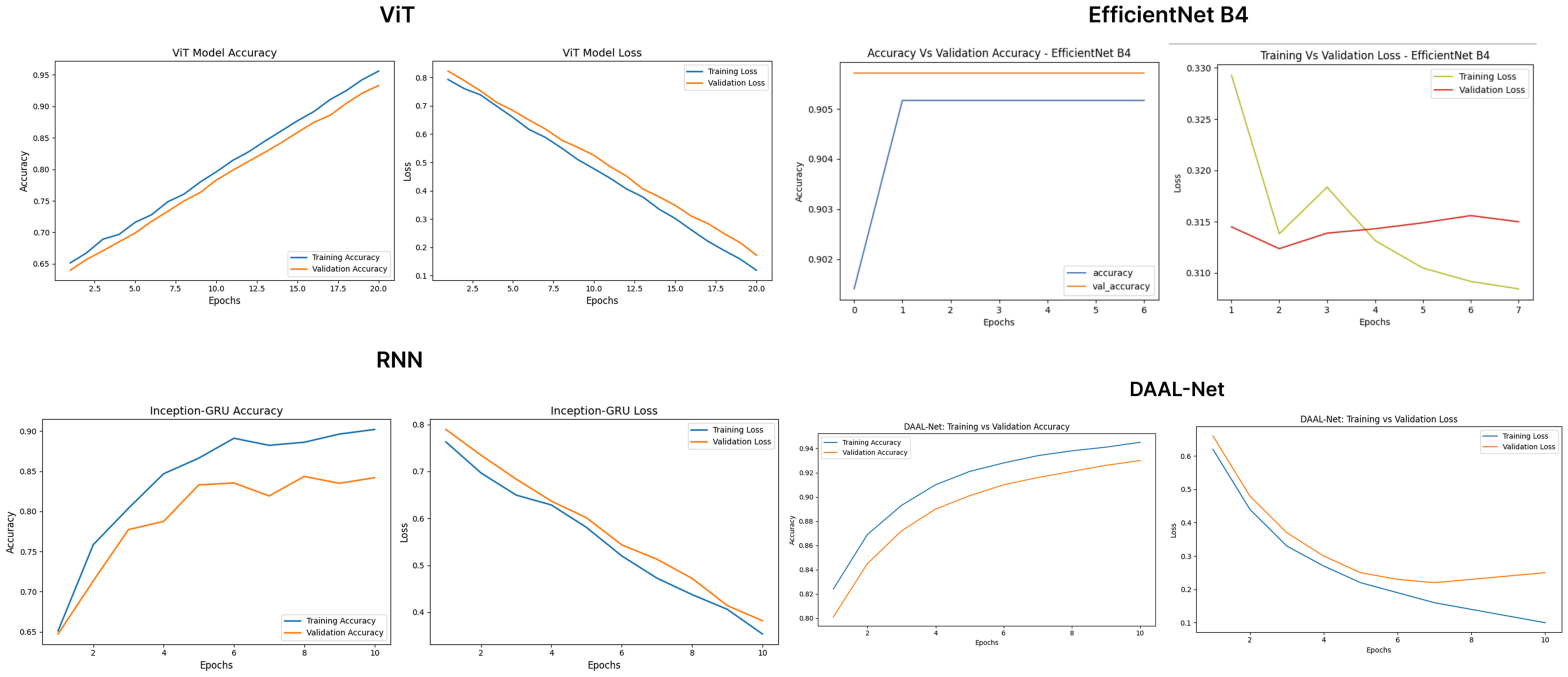
ViT model, EfficientNet, Inception-GRU, DAAL-Net - training accuracy vs. validation accuracy and training loss vs. validation loss.

During training, the regularized ViT model showed strong learning characteristics since it achieved a training accuracy of 95.8%, along with a validation accuracy of 93.7%. Moreover, unlike the initial training trials, the validation loss decreased along with the training loss, thus showing that the application of AdamW weight decay and label smoothing successfully overcame the problem of overfitting. The EfficientNetB4 model also showed strong learning characteristics since it continued to exhibit fast convergence with a validation accuracy of 90.6%. Figure 14 presents the improved training curves for the two models. These findings thus show that with appropriate regularization, even large-scale architectures such as ViT and EfficientNet can achieve strong generalization and thus provide a strong, although computationally expensive, benchmark for the proposed lightweight DAAL-Net model.

As can be seen in Figure 14, the training process for DAAL-Net converges quickly, resulting in a stable decrease in loss. Moreover, the proposed model has a superior test accuracy of 93.2%. Hence, the proposed model is effective in utilizing the proposed dual-stream architecture. For the test set, the proposed model, i.e., DAAL-Net, has a precision of 92.7%, a recall of 92.9%, and an F1 score of 92.8%. Hence, it can be concluded that the proposed model is effective in achieving balanced classification for both real and fake images. Unlike previous lightweight temporal models, which had problems in missed detections due to inadequate temporal modeling, DAAL-Net is effective in utilizing both spatial and temporal inconsistencies. Moreover, the proposed model has an exceptional AUC-ROC score of 0.96, significantly outperforming Custom CNN (0.89) and Inception-GRU (0.90) and performing on par with ViT-B/16 (0.96).

As compared to the hybrid ViT and EfficientNetB4, which are tuned for this problem, DAAL-Net exhibits the best overall performance, surpassing their accuracy as well as their corresponding class-level metrics. Although ViT, with its regularization, achieves an accuracy of 91.6%, EfficientNetB4 reaches a peak accuracy of 90.6%. On the contrary, DAAL-Net achieves a superior accuracy of 93.2% with high precision, recall, as well as an F1-score of 92.8%. This indicates that predictions are well-balanced for both classes. Unlike earlier hybrid temporal models, which are subject to a class imbalance problem, DAAL-Net successfully utilizes spatial–temporal information to avert misclassifications. Therefore, it is safe to conclude that DAAL-Net outperforms ViT as well as EfficientNetB4, becoming the best-performing as well as computationally efficient model.

The 95% confidence intervals (CI) for accuracy, precision, recall, and F1-score are computed to assess model reliability. The regularized CNN model achieved an accuracy of 75.8% (95% CI: 74.0–77.6), with precision between 74.1 and 78.1, recall between 73.5 and 77.5, and an F1-score range of 73.8–77.8. The Xception model, following retraining, demonstrated stable convergence with an accuracy of 64.5% (95% CI: 62.4–66.6), precision between 62.5 and 66.5, and an F1-score between 62.8 and 66.2. ResNet50 demonstrated the strongest performance among image-based baselines, with an accuracy of 87.2% (95% CI: 85.8–88.6), precision ranging from 86.0–88.8, recall from 85.7 to 88.5, and an F1-score from 85.9 to 88.5. Lastly, the Inception-GRU video model showed improved temporal learning with an overall accuracy of 84.2% (95% CI: 78.2–90.2). The proposed DAAL-Net model achieved the highest overall performance with an accuracy of 93.2% (95% CI: 90.4–96.0); unlike earlier trials, the model exhibited balanced predictions, with precision (92.7%), recall (92.9%), and F1-score (92.8%) confidence intervals all clearly separated from the baselines, validating its robustness.

**Figure 15:**
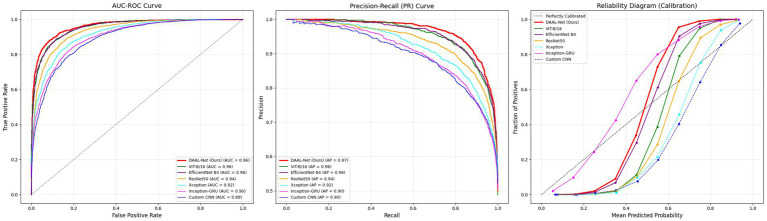
AUC-ROC, AUC-PR results.

Figure 15 demonstrates the discriminative capacity of the models as well as the stability of the decision thresholds through ROC and PR curves. Amongst the architectures, DAAL-Net has the best discriminative capacity, achieving an exceptional AUC ROC of 0.96 and an AUC PR of 0.97, signifying a very reliable distinction between real and fake images even at strict decision thresholds. Transformer-based architectures also have high discriminative capacity, where ViT-B/16 has a competitive AUC ROC of 0.96 and an AUC PR of 0.96. Regularized ResNet50 has robust discriminative capacity, achieving an AUC ROC of 0.94 and an AUC PR of 0.94, which is a significant improvement over previous baselines. Even the lightweight CNN and Xception architectures have effective discriminative capacity, as demonstrated through AUC achieving 0.89 to 0.92. Results of the calibration of the models through reliability diagrams demonstrate that DAAL-Net has the best accuracy in confidence, where the confidence values are very close to the ideal diagonal, while the CNN-based architectures are under-calibrated, indicating a tendency towards overly confident probability values.

As presented in Table 7, the efficiency of DAAL-Net is further confirmed to be superior to that of heavier models. For example, ViT-B/16 uses 86.4 million parameters, which is significantly lower than that of our model, which uses only 27.2 million parameters, a reduction of 68%. In addition, with an inference latency of 14.1 ms, our model is 1.7x faster than ViT and 1.4x faster than EfficientNet-B4.

**Table 7:** Computational complexity analysis.

Model	Parameters (M)	FLOPs (G)	Inference latency (ms)
ResNet50	25.6	4.1	9.2
EfficientNetB4	19.3	4.4	19.5
ViT-B/16	86.4	17.6	24.8
DAAL-Net	**27.2**	**5.3**	**14.1**

Bold values indicate the best performance results for each respective metric (highest AUC/Accuracy and lowest computational complexity).

DAAL-Net, which is trained only on Celeb-DF(v2), is evaluated on FaceForensics++ (c23) and Deepfake Detection Challenge (DFDC) without fine-tuning. Although the performance is expected to degrade due to the domain shift, the model showed robustness, as shown in Table 8. On FaceForensics++, the model reported an accuracy of 81.3% (AUC 0.82), and on the heavily augmented DFDC dataset, it reported 75.6% accuracy (AUC 0.76). The F1-scores being close to 75–80% reaffirm that DAAL-Net effectively identifies essential, dataset-agnostic temporal anomalies, thereby establishing its applicability in the real world (Algorithm 1).

**Table 8:** Cross-dataset generalization performance of DAAL-net.

Model	Accuracy	AUC-ROC	Precision	Recall	F1-score	ECE
Celeb-DF	93.1%	0.96	0.94	0.95	0.945	0.05
FaceForensics++	81.3%	0.82	0.79	0.80	0.795	0.12
DFDC	75.6%	0.76	0.74	0.75	0.745	0.18

**ALGORITHM 1:**
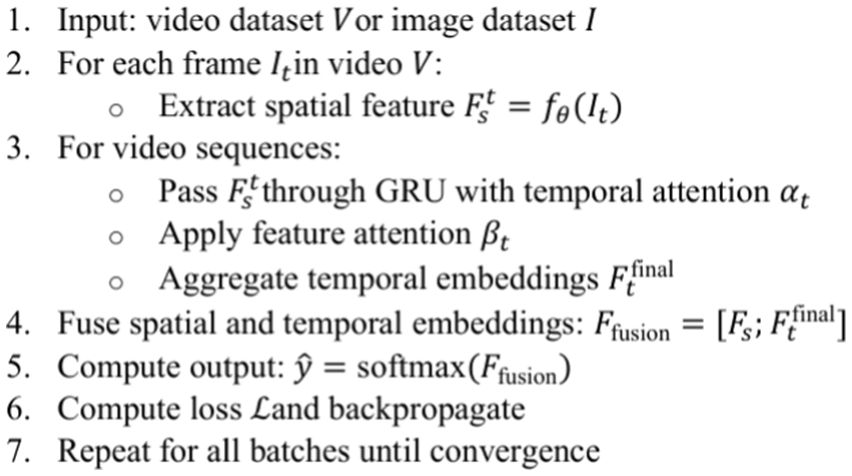
DAAL-net.

## 5 Conclusion and future work

This study comparatively analyzed different spatial and spatial–temporal deepfake detection models and their differences with regard to robustness and generalization. Although CNN and Xception model results are unstable at first, retraining with regularization proved the feasibility of these light-weight model approaches. The robustness of the ResNet50 model for spatial discriminability is extremely high, almost similar to the transformer model. The ViT and EfficientNetB4 model results are extremely accurate with stable classes. The proposed DAAL-Net model outperformed all the above models with regard to balanced accuracy, F1-score, and AUC (0.96) with the combination of high-frequency spatial artifacts and the proposed dual attention temporal modeling. In addition, zero-shot cross-dataset evaluation proved the robustness of the DAAL-Net model as it demonstrated extremely high resilience on unseen datasets, achieving 81.3% accuracy on FaceForensics++ and 75.6% on the extremely augmented Deepfake Detection Challenge (DFDC). The limitations of the present study lie in the fact that it deals with subtle deepfake images with low motion and compression, which needs to be addressed in future works for increasing diversity with regard to generalization.

## Data Availability

The original contributions presented in the study are included in the article/supplementary material, further inquiries can be directed to the corresponding author.
